# Single-Molecule Protein Interactions and Unfolding Revealed by Plasmon-Enhanced Fluorescence

**DOI:** 10.1021/acs.analchem.5c01091

**Published:** 2025-07-19

**Authors:** Roy W.H. Teeuwen, Martina Russo, Maarten Merkx, Peter Zijlstra

**Affiliations:** † Eindhoven University of Technology, Molecular Plasmonics group, Department of Applied Physics and Science Education, 5600 MB Eindhoven, The Netherlands; ‡ Eindhoven University of Technology, Protein Engineering group, Department of Biomedical Engineering, 5600 MB Eindhoven, The Netherlands; § Institute for Complex Molecular Systems, Eindhoven University of Technology, 5600 MB Eindhoven, The Netherlands

## Abstract

Single-molecule characterization of protein interaction kinetics can unravel crucial mechanisms that are averaged out with ensemble-average approaches. However, current approaches based on single-molecule fluorescence are limited in terms of signal brightness and time resolution. We introduce a novel platform to quantify protein–protein interactions at the single-molecule level using plasmon-enhanced fluorescence microscopy. We illustrate the power of this approach using PDZ protein that is conjugated to plasmonic particles using a novel DNA-mediated hybridization method that provides spatial and orientational control over the proteins’ immobilization. Single-molecule kinetic studies uncover heterogeneities in the interaction where a subpopulation of events exhibits a distinct bound-state-lifetime not observed before. This new method also enables the study of urea-mediated unfolding and refolding using binding kinetics as readout. The bound-state lifetime was found to be independent of urea concentration, implying a simple two-state unfolding model. In addition, we find that the folding is entirely reversible for the immobilized PDZ, in contrast to solution-phase unfolding that results in aggregation. Altogether, our results present single-molecule plasmon-enhanced fluorescence as a new and powerful method to monitor transient protein–protein interactions and protein folding on short time scales.

## Graphical abstract

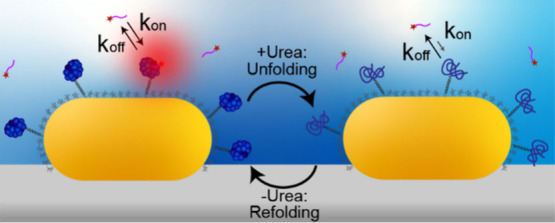

## Introduction

Protein–protein interactions (PPIs) play a pivotal role in human physiology. PPIs are often transient, such as those involved in signal transduction, regulatory cell processes, and chaperone mediated protein folding. Disruptions in such highly orchestrated interaction networks are involved in various diseases, including cancer and neurodegenerative disorders.^1–3^ To understand PPIs, it is important to characterize the underlying kinetic behavior. Historically, the kinetics of PPIs have been studied using ensemble-average techniques such as surface plasmon resonance assays and bilayer interferometry. However, these techniques are less suitable for studying interactions with high association and/or dissociation rate constants. Additionally, due to their ensemble-average nature, information regarding heterogeneity in the interaction pathways is hidden.

A promising avenue is the use of single-molecule fluorescence microscopy.^4^ Herein, either one or multiple interaction species studied are labeled with a fluorophore, enabling the single-molecule detection of interaction kinetics if one of the proteins is immobilized. Single-molecule pulldown assays and point accumulation for imaging nanoscale topography (PAINT) can for example be used to study binding kinetics and stoichiometries.^5,6^ Förster resonance energy transfer (FRET) microscopy utilizes the distance-dependent energy transfer between donor and acceptor to visualize protein interactions and conformational dynamics.^7,8^

Despite its power to reveal interaction pathways, single-molecule fluorescence microscopy comes with limitations. First, the fluorescently labeled proteins that are not engaged in the PPI contribute to the background because they diffuse through the excitation volume. This limits the signal-to-background ratio of the measurement and complicates data analysis. Moreover, this limits the maximum concentration of labeled protein to the low nanomolar regime to limit the fluorescence background. The use of zero-mode waveguides (ZMWs), in which fluorophores are only excited within nanoscopic wells, can circumvent these issues.^9–11^ While ZMWs strongly reduce the background, they do not strongly enhance the fluorescence of fluorophores in the aperture.

One approach to simultaneously suppress background and enhance the fluorescence brightness lies in the use of nanoplasmonic structures, such as gold nanoparticles (GNPs). Upon illumination with an excitation laser the conduction electrons of the particles oscillate resonantly at a frequency that depends on the size, shape, and material of the particle.^12^ These oscillating electron clouds (plasmons), give rise to highly confined electric near-fields around the GNPs. Within these fields, the fluorescence of single emitters is greatly enhanced due to excitation and emission enhancement.^13,14^ Besides offering a potent approach to facilitate a high signal-to-background, the enhanced fluorescence achieved through nanoplasmonics can also be used to enhance the temporal resolution of the measurements toward the microsecond time scale.^15^ Grabenhorst et al. reported on a system where DNA origami was used to form a plasmonic hotspot between 2 plasmonic nanoparticles.^16^ This system enabled them to follow the fast interaction between two intrinsically disordered proteins on a microsecond time scale. However, the complex nature of the assembly limits the throughput, complicating the acquisition of sufficient statistics to quantify PPIs and their heterogeneity.

Our group recently reported a simpler design, utilizing immobilized single gold nanoparticles as plasmonic nanoantennas to study DNA interactions with high fidelity.^17–19^ Transient interactions between the oligonucleotides on the particles and the fluorescently labeled oligonucleotides in solution resulted in plasmon-enhanced fluorescence bursts on the nanoparticles, permitting single-molecule characterization of binding kinetics. Despite their promise in quantification of DNA interactions, there remains an urgent need for a platform to quantify ubiquitous PPIs with sufficient statistics and signal brightness to uncover potential PPI heterogeneity.

Here, we developed a plasmon enhanced fluorescence-microscopy approach to characterize single-molecule PPI interactions with high statistics. We employ a DNA-based protein conjugation method to immobilize one of the binding partners in an oriented and distance-controlled manner to the GNPs. Our single-molecule approach uniquely allows for the quantification of both the association and dissociation rate constants of a model PDZ protein. The high signal-to-background ratio combined with the high temporal resolution reveals that the dissociation process is heterogeneous and exhibits two distinct time-constants. In addition, we demonstrate the ability to quantify urea-mediated unfolding and refolding using binding kinetics as readout. We find that the bound-state lifetime is independent of urea concentration, implying a simple two-state unfolding model. In addition, we find that the folding is entirely reversible for the immobilized PDZ, in contrast to solution-phase unfolding that results in aggregation. Altogether, the presented results pave the way toward the single-molecule characterization of fast PPIs and their modulation by protein unfolding and refolding.

## Methods

### Protein–DNA Conjugation

MBP-PDZ proteins containing a single cysteine residue were expressed in BL21­(DE3) cells and subsequently purified using both nickel affinity chromatography and Strep-Tactin affinity chromatography. Purified proteins were fluorescently labeled with ATTO 532-NHS and then reduced using TCEP. Next, a PD10 desalting column was used to remove both the unreacted dye and TCEP. Amine-modified DNA strands were reacted with sulfo-SMCC to provide them with a maleimide handle. Excess cross-linker was removed using salt precipitation, after which the DNA was reacted with the proteins to obtain protein–DNA conjugates. Excess DNA was removed through Strep-Tactin column chromatography, after which the buffer was exchanged using a PD10 column, and the final product was reconcentrated using a 10 kDa centrifugal amicon column.

### Gold Nanoparticle–Protein Conjugation

Citric acid stabilized gold nanoparticles (40 by 80 nm^2^) were coated with single-stranded thiolated DNA strands in a low pH (3.0) citrate buffer. Excess DNA was removed through 4 ultracentrifugation steps, after which the DNA-functionalized proteins were added. Following DNA hybridization, and thus formation of the GNP-protein conjugates, another 4 centrifugation steps were used to remove excess protein-DNA conjugate.

### Fluorescence Microscopy

Samples for microscopy measurements were prepared by first sonicating Menzel #1.5 glass slides in methanol for 15 min. Sonicated slides were blown dry using nitrogen gas and further cleaned through 1 min of plasma treatment. Well-stickers were added on top of the cleaned glass slides, after which the functionalized GNPs were drop-casted into the wells and incubated for at least 5 min to allow spontaneous immobilization. Samples were then imaged through TIRF microscopy using a 637 nm laser and 60× 1.49 NA oil objective. In each measurement, ATTO 655-labeled target peptide was added, allowing the PDZ-peptide binding interactions to be visualized as clear fluorescence bursts on the particles.

## Results

In this study we utilize single-crystalline GNPs (nanorods) to probe protein–protein interactions at the single-molecule level. To facilitate single-molecule studies the particles are immobilized onto a glass coverslip to enable the imaging of single nanoparticles. Before immobilization, we conjugated protein (PDZ) to the nanoparticles using a solution-phase protocol. We initially tried direct conjugation of PDZ to the gold nanorods via a cysteine linker. However, this resulted in proteins that showed no peptide binding, presumably due to partial protein denaturation. We then developed a DNA-mediated conjugation approach. This approach, through the rigid nature of double-stranded DNA, allows for control over the time-average distance between the proteins and the GNPs. This controlled distance is crucial, as it prevents a direct interaction between the protein and the gold, thereby preventing denaturation. Moreover, it still allows the proteins to be placed close to the gold nanorods, which facilitates potent fluorescence enhancement of the fluorescently labeled binding partners (Supplementary Figure 1).

We used a model system consisting of an Erbin PDZ protein that transiently interacts with a peptide-binding partner. Previous research reported fast association and dissociation kinetics for this PDZ interaction with subsecond bound-state lifetimes.^20^ PDZ proteins were expressed through bacterial expression, with an N-terminal maltose binding protein domain to improve yields. Since neither the utilized PDZ domain nor MBP contains natural cysteines, a single cysteine was introduced between the protein domains as a site-specific functional handle to obtain reproducible oriented conjugation. Combined with the programmable particle-protein spacing facilitated by the DNA linker, this oriented conjugation approach minimizes the effects of protein immobilization on the binding kinetics. For the conjugation reaction, a bifunctional cross-linker (sulfo-SMCC), containing both a thiol-reactive maleimide group and amine-reactive sulfo-NHS ester group, was used to couple the amine-modified DNA oligonucleotides to the cysteine residue (Figure 1A). Excess oligonucleotides were washed away using Strep-tactin affinity chromatograph. Although 50% of the proteins in the conjugation mixture did not react with DNA, unreacted proteins were not removed from the final product as they were not expected to interfere with downstream protein-GNP functionalization steps (Supplementary Figure 2). Before DNA-conjugation the construct was labeled with a green dye (ATTO532-NHS) to enable quantification of the number of proteins per particle at a later stage. Note that the green fluorophore does not interfere with downstream measurements because PPIs were quantified at near-infrared wavelengths.

**Figure 1:**
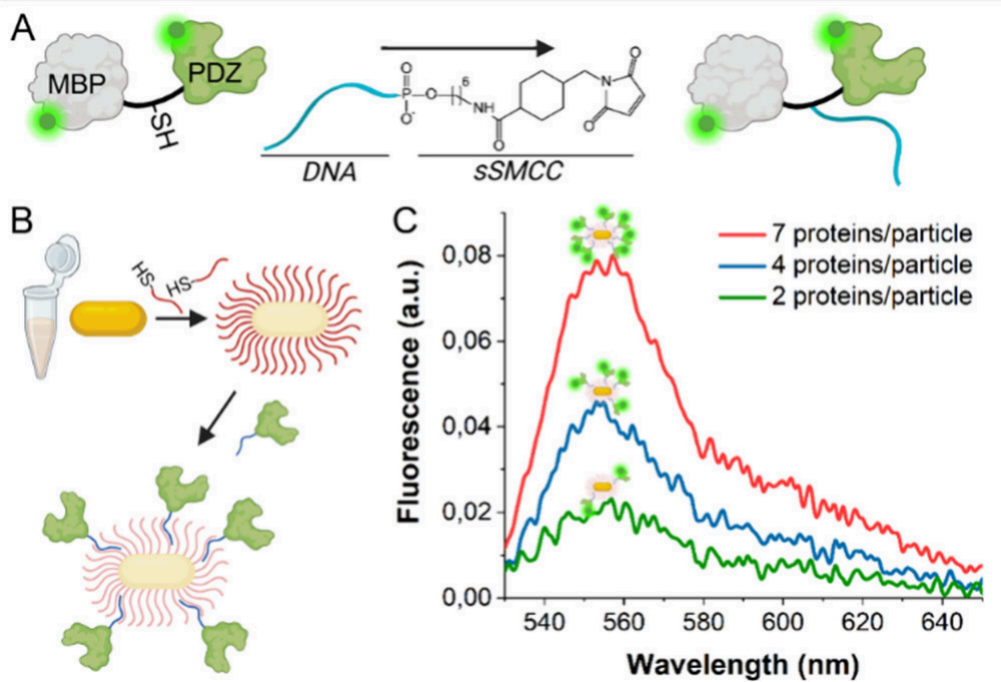
Solution-phase functionalization of gold nanoparticles. (A) overview of the site-specific protein–DNA labeling method. The MBP-PDZ protein contains a single cysteine residue. Through incubation with a maleimide functionalized DNA (DNA-amine functionalized with cross-linker sulfo-sMCC), the DNA is attached to the protein at a predetermined position with 1:1 stoichiometry. (B) Gold nanoparticles are first functionalized with a single-stranded oligonucleotide monolayer. Next, the protein–DNA conjugate, of which the oligonucleotide is complementary to that on the particles, is added (MBP domains not drawn). (C) Protein grafting densities can be controlled by varying the added PDZ-DNA concentrations (added concentrations doubled between the samples of the green and blue, and blue and red curves), and quantified through fluorescence readout (using fluorescently labeled PDZ).

Solutions with GNPs were functionalized with a monolayer of single-stranded oligonucleotides through thiol-gold interactions, allowing for protein-GNP grafting through DNA hybridization (Figure 1B). All conjugations were performed using 40 by 80 nm^2^ gold nanorods, as these GNPs possess sharp localized surface plasmon resonance peaks around 650 nm, enabling strong enhancement of the used fluorophore (ATTO 655) in downstream PPI measurements. Afterward, excess protein–DNA conjugates were washed away, and grafting was assessed through a combination of UV–vis spectroscopy and platereader-based fluorescence readout. UV–vis measurements were used to assess colloidal stability (Supplementary Figure 3), and to measure GNP concentrations. Final protein-GNP conjugation products showed absorption spectra close to those of bare GNPs, highlighting their maintained colloidal stability. Fluorescence spectroscopy (platereader) measurements (Figure 1C) were used to quantify the samples’ protein concentrations, as their fluorescence emission at 552 nm directly correlate to the concentration of ATTO 532 labeled PDZ. As such, a calibration curve correlating known concentrations of ATTO 532 labeled PDZ to the corresponding fluorescence intensity at 552 nm (Supplementary Figure 4) enabled us to infer each conjugation sample’s protein concentration based on their emission at the same wavelength. Together, the GNP and protein concentrations were then used to determine the average number of proteins per particle for each sample. Overall, this approach yielded a highly reproducible method to functionalize the gold nanoparticles, in which the average number of immobilized proteins per particle linearly scaled with the added protein–DNA conjugate concentration (Figure 1C). The highest protein density we used in this study (7 per particle) is lower than the theoretical maximum dictated by steric effects. Assuming a random sequential absorption process we find a maximum of ∼200 proteins per particle because 40% of the GNPs’ surface area can be covered. However, increasing the added protein–DNA concentration during functionalization will yield higher densities if desired. Moreover, negative controls (DNA-coated GNPs incubated with proteins without a DNA handle) showed hardly any protein attachment, highlighting the specificity of the approach (Supplementary Figure 5).

To determine the single-molecule binding kinetics of the PDZ-peptide interactions, the protein-decorated particles were sparsely immobilized on glass coverslip and imaged on a fluorescence-microscopy setup. The fluorescently labeled peptide transiently binds to PDZ, resulting in strong plasmon-enhanced fluorescence bursts on the gold nanoparticles (Figure 2A). We estimate an enhancement factor of 6–8 times, which is obtained by comparting the intensity of the top 10% brightest plasmon-enhanced fluorescence events on the gold nanoparticles to the average brightness of nonspecific interactions with the glass coverslip away from the particles (Figure 2B). This enhancement factor lays slightly lower than predicted based on simulations (Supplementary Figure 1). Since ATTO 655 is quenched by electron donors such as guanine, we hypothesize that this may partially be caused by the GNP’s DNA coating. Nevertheless, the correspondingly improved signal-to-noise enables short exposure times (20 ms in this study) with an average signal-to-noise ratio (SNR) of 197, permitting quantification of short-lived interactions with high fidelity.

**Figure 2:**
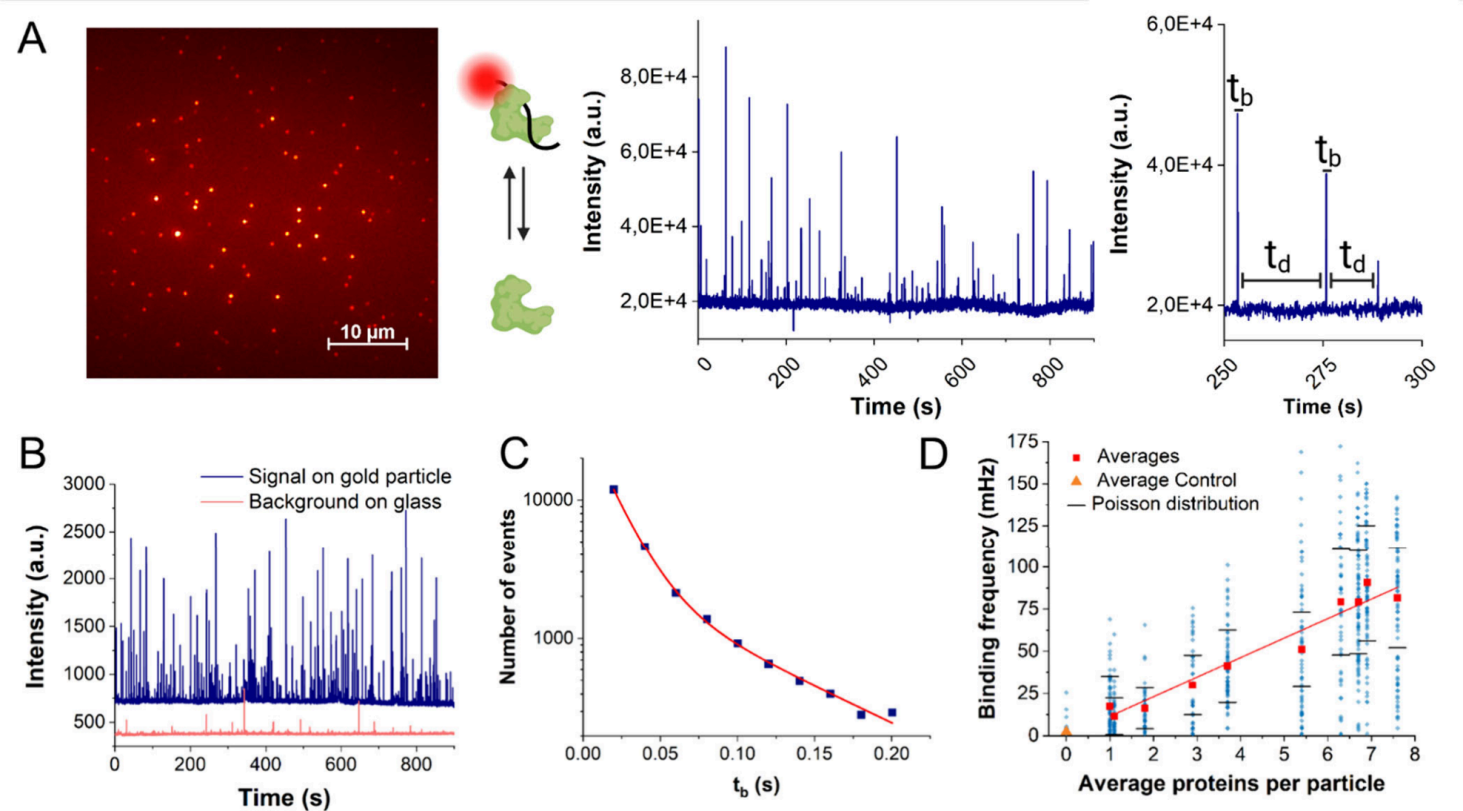
Characterization of the PDZ-peptide binding interactions. (A) Typical microcopy FOV of the immobilized gold nanoparticles. Each FOV (∼ 43 μm by 43 μm) typically contains around 100 stochastically immobilized particles, yielding average interparticle distances of several micrometers. A time trace of one of the gold nanoparticles is shown, on which the peptide–protein binding events can be seen as fluorescence bursts. An expanded region of the time trace showcases a few examples of individual bright-times and dark-times. (B) Typical signals obtained from peptide binding on a gold nanorod (blue) and on the glass (red). (C) Plot depicting the distribution of bright times (bound-state lifetimes) of the protein-peptide interactions, based on all recorded particles. The red line represents a double exponential decay fit. (D) Correlation between the average number of proteins per particle, and the fluorescently labeled peptide’s binding frequency. Blue data points represent individual particles within a sample, and red square data points the mean values per sample. The yellow triangular data point represents the mean event frequency of negative controls (DNA-coated particles without proteins attached). The linear fit has an intercept and slope of 0 and 12 mHz/protein, respectively. Error bars represent the effect of each sample’s Poisson distribution of the number of proteins per particle on the average binding frequencies.

When assessing the fluorescence time traces, the duration of the fluorescence events (“bright times”, *t*_b_) directly correlate to the bound-state lifetimes of the protein-peptide interactions ( $k_{off}=\frac{1}{t_{b}}$ ).^21^ The overall distribution of the bound-state lifetimes is double exponential (Figure 2C), indicating that the interaction involves two characteristic processes. The double-exponential distribution was also observed in the statistics obtained from a single particle (Supplementary Figure 6), eliminating particle-to-particle variations as its root cause. On the other hand, experiments on DNA–DNA interactions using the same method revealed a single-exponential distribution of bound-state lifetimes expected for a molecular process with a single rate constant.^15^ This confirms that the double-exponentiality originates from the PPI itself.

From a double-exponential fit we extracted characteristic bound-state lifetimes of 16 ± 1 and 83 ± 16 ms, corresponding to *k*_off_ values of 61 ± 5 and 12 ± 3 s^–1^. The slow dissociation rate constant is similar to previously reported values for the same interaction with immobilized peptides (*k*_*off*_ ≈ 6.3 s^–1^).^20^ This study employed an integration time of 60 ms, limited by the signal-to-noise ratio of regular single-molecule fluorescence studies, and therefore did not fully resolve the fast component that is uncovered using our approach (20 ms integration). The underlying molecular mechanism leading to two distinct subpopulations is not known, but suggests the presence of two previously unresolved conformations for the protein-peptide complex. However, due to the significantly less frequent occurrence of the events corresponding to the longer bound state lifetimes, we cannot yet conclude whether this is caused by an intermediate state or by a multistate binding mechanism.

As described in ref 17, the uncertainty in the counted number of binding events is determined by the total number of events N, and equals √N as dictated by Poisson statistics. This error bar is of similar size or smaller than the data points in Figure 2C. Additional imprecision due to the occurrence of double events can be neglected here because we use a low concentration of fluorescent probes, leading to a low event rate. The main dispersion in our measurements therefore comes from particle-to-particle differences in the density of conjugated proteins, displayed in Figure 2.D.

In order to quantify association kinetics and related particle-to-particle differences, analysis is based on the average dark time (*t*_*d*_) in between events (unbound-state lifetime). Together with the number of proteins per particle (*n*), and the concentration of the labeled peptide solution (*c*_*i*_), it follows that $k_{on}=\frac{1}{t_{d}c_{i}n}$ .^21^ The binding event frequency’s dependency on the number of proteins per particle was assessed through several measurements, using particle preparations with different average numbers of proteins per particle (Figure 2.D). Herein, a control sample (DNA decorated, 0 proteins per particle) showed nearly no events (2 mHz), indicating a low probability of unspecific binding of the peptides to the particles, with a therefore correspondingly minimal effect on the measurements. For the particles functionalized with proteins, event frequencies show a relatively large spread, owing to the combined Poisson distributions of the number of proteins per particle, and the peptide binding event frequencies. Nevertheless, since every nanoparticle can be seen as an individual sensor, and each sample contains a significant number of particles, ample statistics were gathered to observe a clear linear behavior between the average number of proteins per particle and event frequencies. Using the corresponding linear fit, a *k*_*on*_ = (1.2 ± 0.1) × 10^7^ M^–1^ s^–1^ is obtained that is substantially higher than previously reported based on PAINT experiments (*k*_*on*_ ≈ 3.6 × 10^5^ M^–1^ s^–1^).^20^ This difference can be explained by the improved SNR achieved through plasmon enhancement. Dim fluorescence signals that would have been lost in unenhanced studies can now readily be detected, giving rise to more accurate event detection, and thus, a more representative *k*_on_ value. Based on the determined kinetic constants, the overall affinity of the interaction ( $K_{d}=\frac{k_{off}}{k_{on}}$ ) is found to be 1.0–5.1 μM (based on the slower and faster *k*_*off*_ subpopulations respectively). This *K*_*d*_ value corresponds well with the ensemble-average measured affinity previously reported for Erbin PDZ toward a peptide harboring the same N-terminal binding sequence (DTWV, *K*_*d*_ = 2.8 μM),^22^ and is in good agreement with our own ensemble-average fluorescence polarization measurement (Supplementary Figure 7).

Next, we employed the platform to monitor protein unfolding and refolding. To this end, we exposed the protein-coated GNPs to increasing concentrations of urea, which is known to efficiently unfold various members of the PDZ protein family at low molar concentrations.^23,24^ As predicted based on these literature reports, the fluorescence time traces of the PDZ-peptide measurements showed a clear decrease in binding frequency when an excess of urea was added (Figure 3A). After validating that the observed event rate decrease was not caused by protein dissociation from the GNPs (Supplementary Figure 8), we next characterized the concentration dependency of this urea-mediated PDZ unfolding (Figure 3B). The protein-peptide binding frequency, and thus PDZ conformational integrity, clearly decreases in a urea concentration-dependent manner. Although a slight increase in the average protein-peptide binding event frequency is observed at 0.2 M urea, this effect is only minor and falls well within the provided Poisson distribution. Correspondingly, we do not conclude that such urea concentrations enhance the binding interaction to a significant extent. However, A significant decrease of the binding event frequency is observed between 0.8 and 1.6 M urea, suggesting that urea starts to efficiently unfold the proteins. When removing the denaturant from a sample preincubated with 6.4 M urea (Figure 3B) binding frequencies recovered toward initial levels, indicating efficient refolding of PDZ within ∼1 min required for fluid exchange and the subsequent initiation of imaging.

**Figure 3:**
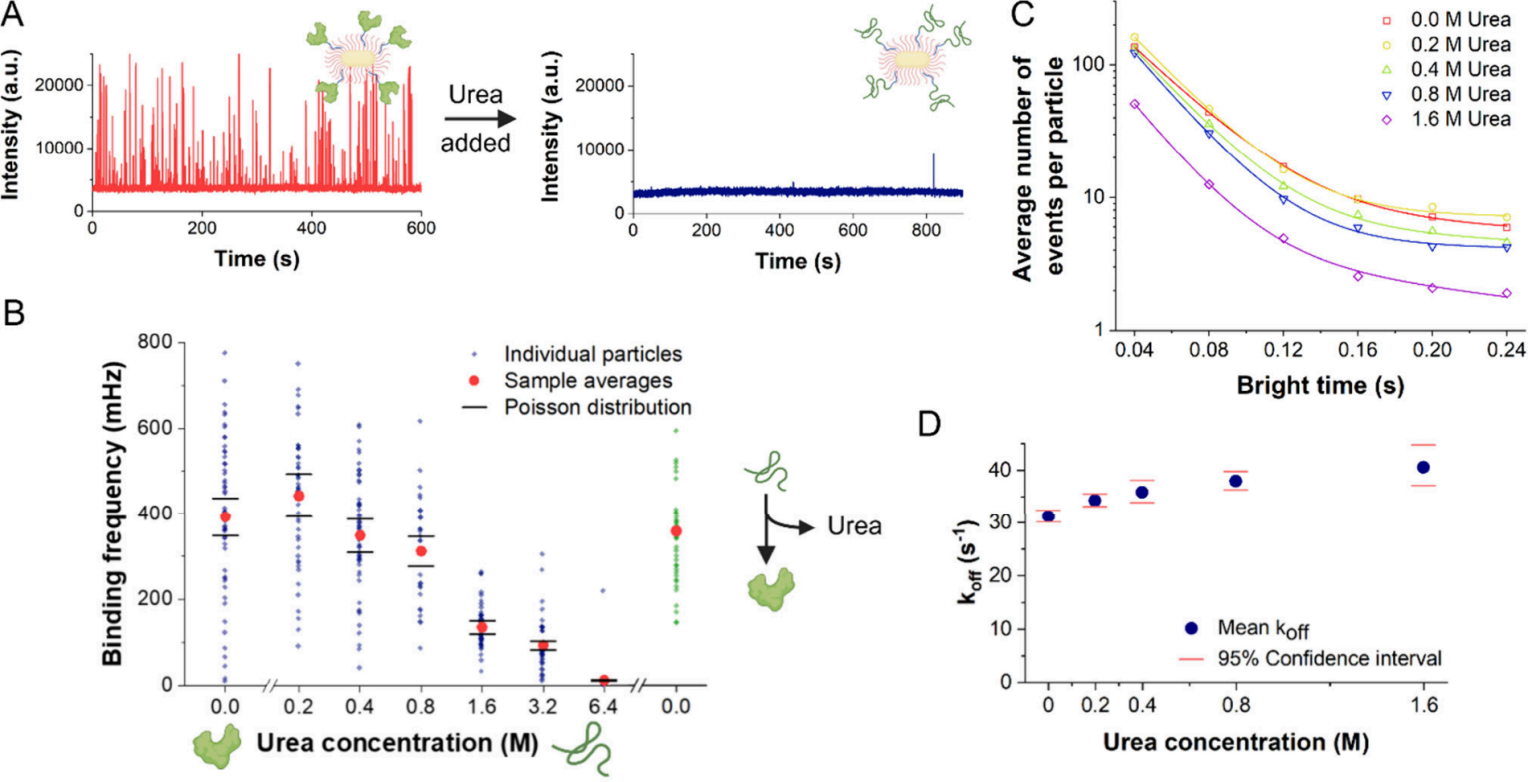
Urea-mediated PDZ denaturation. (A) Addition of urea terminates the occurrence of PDZ-peptide interactions as seen on the examplatory fluorescence timetraces of a particle before and after addition of 4.0 M urea. (B) Left/blue data points: PDZ-peptide binding event frequencies at different urea concentrations. To prevent cumulative and/or time-mediated influences, measurements at each concentration were performed with a fresh sample. Error bars represent the effect of each sample’s Poisson distribution of the number of proteins per particle on the average binding frequencies. Right/green data points: PDZ-peptide binding event frequencies of a similar sample, after incubation at 6.4 M urea and subsequent removal thereof. (C) Bright-time distribution of the PDZ-peptide binding events at different urea concentrations. Lines represent double exponential fits. (D) Dissociation rate constants per urea concentrations, corresponding to the short t_b_ value of each double exponential fit. The 95% confidence interval is obtained by propagating 1.96 SE of the corresponding t_b_ fits.

We also quantified how the presence of denaturant affects the distribution of bound-state lifetimes. Bound-state lifetimes of the remaining protein-peptide interactions did not significantly change for increasing urea concentrations (Figure 3.C), as observed based on the similar bright time distributions. Corresponding *k*_*off*_ values (based on the dominant short characteristic lifetimes of the double exponential fits) show no significant deviations (Figure 3.D). The results are consistent with a simple two-state model in which the protein is in equilibrium between a binding-competent folded state and a binding incompetent unfolded state. Herein, the protein may be properly folded, undergoing peptide binding events, or in an unfolded state in which it no longer binds peptides. Altogether, these unfolding studies highlight the strength of the developed system, enabling not only readout on ensemble-average protein stability, but also the underlying kinetic behavior and folding state.

## Conclusion and Discussion

Within this work, we have presented a modular approach to form protein-GNP conjugates, with control over both protein orientation as well as spacing from the GNP’s surface. Functionalized particles enabled plasmon-enhanced fluorescence-based characterization of protein-peptide interactions with high fidelity. The system was used to map the association and dissociation of a PDZ-peptide interaction. A key observation included the presence of a subpopulation of protein-peptide interactions with a short bound-state lifetime not observed by previous researchers. This demonstrates the clear benefits of employing plasmon enhanced fluorescence, enabling measurement at high temporal resolution while maintaining a high SNR. Lastly, the developed platform enabled the study of PDZ’s urea-mediated denaturation. Clear urea-dependent unfolding was observed. Unlike studies in bulk,^25^ unfolding of immobilized PDZ was completely reversible, presumably due to suppression of aggregation. Moreover, peptide dissociation kinetics were not affected by the presented urea concentration, suggesting a simple two-state folding model.

The DNA hybridization-based GNP functionalization approach provides a reproducible and modular means to conjugate functional proteins to GNPs. Although we introduced a single cysteine amino acid in the protein to perform the protein–DNA conjugations, this approach may not be feasible for proteins naturally containing surface-exposed cysteines. In such cases, we envision similar site-specific DNA conjugations using mutagenically introduced noncanonical amino acids with other functional handles, such as azides.^26^ In our current study, fluorescence microscopy measurements of the protein-peptide interactions were performed with an exposure time as low as 20 ms. However, owing to the high SNR, further decreases in the exposure time are well possible, enabling the study of even faster interactions. Altogether, the approach may unravel novel insights regarding various assets of fast PPI kinetics, as well as protein folding and unfolding mechanics. Among others, these insights may prove to be of crucial importance in gaining understanding of fundamental protein mechanisms and protein-associated diseases.

## Supplementary Material



## Notes

Materials and methods, simulation distance dependence of fluorescence enhancement ATTO655 in close proximity to nanoparticle, SDS-PAGE gel of PDZ-DNA conjugation, absorption spectra of GNP-protein conjugates, calibration curve utilized to calculate the average number of proteins per GNP, fluorescence spectra of GNP-protein conjugations including negative controls, bright time distribution plot of 2 individual GNPs, fluorescence polarization curve for PDZ-peptide interaction, and validation of stability GNR-protein conjugation at urea exposure (PDF)
