# Supplementary material for: Single-Molecule Protein Interactions and Unfolding Revealed by Plasmon-Enhanced Fluorescence
Source: Anal Chem. 2025 Jul 19;97(29):15651–7. doi: 10.1021/acs.analchem.5c01091 (PMC12311895; doi:10.1021/acs.analchem.5c01091)
Supplement: Supplementary file 1 [file ac5c01091_si_001.pdf]

Supplementary Information

Single-molecule protein interactions and unfolding revealed by plasmon-enhanced fluorescence

Roy W.H. Teeuwen[1,3], Martina Russo[1,3], Maarten Merkx[2,3]*, Peter Zijlstra[1,3]*

1: Eindhoven University of Technology, Molecular Plasmonics group, Department of Applied Physics and Science Education, 5600 MB Eindhoven, The Netherlands. 2: Eindhoven University of Technology, Protein Engineering group, Department of Biomedical Engineering, 5600 MB Eindhoven, The Netherlands. 3: Institute of Complex Molecular Systems, Eindhoven University of Technology, 5600 MB Eindhoven, The Netherlands

Contents;

Materials and methods………………………………………………………………………………………. 2-3

S. Fig. 1. Simulated fluorescence enhancement ATTO 655 close to 40x80 nm^2 GNPs…………………. 4
S. Fig. 2. SDS-PAGE analysis of protein-DNA conjugation……………………………………………. 4
S. Fig. 3. Absorption spectra of protein-GNP conjugation……………………………………………… 5
S. Fig. 4. Calibration curve fluorescence intensity - ATTO 532-labeled PDZ concentration …………... 5
S. Fig. 5. Fluorescence spectra protein-GNP conjugation……………………………………………….. 6
S. Fig. 6. Single-particle bright time distributions protein-peptide binding events……………………… 6
S. Fig. 7. Fluorescence polarization curve PDZ-peptide interaction…………………………………….. 7
S. Fig. 8. Fluorescence readout of protein-GNP conjugate stability when exposed to urea…………….. 8

Materials and methods

Oligonucleotides and peptides

All oligonucleotides were ordered from Integrated DNA Technologies Europe, with HPLC purification.

Table S.1. overview of used oligonucleotides using throughout this study.

Description:	Sequence:
Conjugated to proteins	/5AmMC6/TA GAT GTA TTA TTG GAA GCG T
Immobilized on gold nanorods	/5ThioMC6-D/CA TCA TCA TAC GCT TCC AAT AAT ACA TCT A

The Atto-655 labeled peptide (H-[C(ATTO655)]GAGRGSIDTWV-OH) was purchased from SB Peptide with 99% purity.

Protein expression and purification

PDZ was expressed as a fusion protein, containing an N-terminal Maltose Binding Protein (MBP) domain to aid in expression yield. The corresponding pET28a plasmid was ordered from Genscript. Proteins were expressed in *E. coli* BL21(DE3) cells, through growing a 500 mL culture in LB medium until OD600 = 0.6 (250 rpm, 37 °C), subsequent addition of 250 µM IPTG, and overnight incubation at 250 rpm, 20 °C. Cells were isolated through centrifugation, and lysed over 1 hour using 5 mL of BugBuster protein extraction reaction per gram of bacterial pellet. Lysed cells were centrifuged, and the supernatant was filtered over a 0.2 µm syringe filter. The obtained protein solution was first purified using nickel affinity chromatography (His-Bind Ni-NTA resin), followed by Strep-Tactin chromatography (Strep-TactinXT 4Flow High Capacity resin). The purified protein was snap-frozen in liquid nitrogen, and stored at -70 °C until further use.

Protein fluorescent labeling and reduction

The purified protein was first re-buffered to phosphate buffered saline (PBS) using PD10 desalting columns (Cytiva), after which the volume was reduced using an 10 kDa 4 mL amicon spin filters. A 0.5 mL labeling reaction was performed using 25 v/v% 100 mM sodium bicarbonate buffer (pH 8.75), protein towards a final concentration of 100 µM, Atto-532-NHS towards a final concentration of 200 µM (using a 10 mM stock dissolved in anhydrous DMSO), and additional PBS towards a final volume of 0.5 mL. The labeling reaction was incubated for 3 hour at room temperature. After fluorescent labeling, TCEP was added to a final concentration of 4 mM to reduce disulfide bonds between proteins, rendering all proteins' cysteine residues available for DNA conjugation.. Following another 2 hour incubation, excess dye and TCEP were removed using a PD10 desalting column (100 mM sodium phosphate buffer (pH 7.0).

Protein-DNA conjugations

The amine-modified oligonucleotide was incubated with the sulfo-SMCC crosslinker in separate 80 µL small-scale reactions with 125 µM oligonucleotide, 1.2 mM sulfo-SMCC, 37.5 v/v% PBS, 50 v/v% DMSO, 12.5 v/v% milliQ water (2 hours at 250 rpm, room temperature). Excess crosslinker was removed using ethanol precipitation. Each tube received 240 µL 100% ice-cold ethanol + 8 µL 5 mM NaCl, and stored at -20 °C for 75 minutes. Precipitated DNA was pelleted using centrifugation (15 min. at 4 °C and 14000 rpm), and re-dispersed in 240 µL of fresh 100% ice-cold ethanol + 8 µL 5 M NaCl. After identical incubation and centrifugation, pellets were dissolved in 200 µL ice-cold 95% ethanol. Tubes were immediately centrifuged, after which the supernatants were pipetted off and pellets were fully dried and stored at -20 °C until further use.

Fluorescently labeled & reduced protein was incubated with the sulfo-SMCC functionalized oligonucleotide. For this, 2.5 nmole protein was added to each 10 nmole oligonucleotide tube, and volumes were adjusted to 200 µL using additional 100 mM NaPi (pH 7.0). Following 2 hour incubation (RT), all reactions were applied to- and purified with- a Strep-Tactin column to remove excess oligonucleotide. The eluate's volume was reduced through a 4 mL 10 kDa amicon filter, after which the buffer was exchanged towards 25 mM Tris (pH 8.0) + 37.5 mM NaCl through a PD10 desalting column. Following a final amicon-based concentration step, the protein-DNA conjugate was aliquoted, snap-frozen in liquid nitrogen, and stored at -70 °C until further use.

GNP-protein conjugations and quantifications

40x80 nm citrate capped gold nanorods (NanoPartz) were functionalized with thiol-modified oligonucleotides in small reaction scales containing 10 µL 50 µM oligonucleotide, 10 µL 10 mM TCEP, 10 µL 100 mM citrate buffer (pH 3.0) + 1M NaCl, 25 µL mQ, and 150 µL OD 2 citrate particles (at 650 nm, 1 cm pathlength) diluted in milliQ water in a DNA LoBind Eppendorf tube. Reactions were incubated for 1 hour, after which they were purified through centrifugation (4-

5 minute steps at 5000 rpm each). After each centrifugation step, supernatants were discarded, and pellets were re-dissolved (200 µL of 25 mM HEPES (pH 7.4) after the first spin step, 200 µL of milliQ after step 2 and 3, and 150 µL of 10 mM HEPES (pH 7.4) + 0.0025 w/v% Tween20 + 1 mM $MgCl_2$ after step 4.

For each reaction, 5.0 µL of protein-DNA conjugate was added to a 150 µL particle solution as prepared above. Added protein-DNA concentrations varied from 250 pM to 2 µM depending on the desired number of proteins per particle. Reactions were either incubated for 2 hours at room temperature (~21 °C), or overnight in the fridge (4 °C). After incubation, 1.5 µL of 20 w/v% BSA in PBS was added to each sample, following an additional 15 minutes of incubation. Next, samples were washed using 4 centrifugal steps (5 minutes at 5000 rpm and 4 °C each). After each spin step, supernatants were removed, and particles were re-suspended using 10 mM HEPES pH 7.4, 0.0025 w/v% Tween20, 1 mM $MgCl_2$, 0.2 w/v% BSA (150 µL after step 1-3, 75 µL after step 4).

Functionalized gold nanoparticle concentrations were determined through absorption measurements on a Thermo Scientific Nanodrop 2000 apparatus. Fluorescence spectroscopy-based protein quantifications were performed through fluorescence measurements on a Varioskan Lux platereader (500 nm excitation, 12nm bandwidth, low dynamic range, 1000 ms per well).

Sample preparation for microscopy measurements

Glass microscopy slides (Menzel #1.5 24*40 mm) were sonicated in MeOH for 15 minutes, and blown dry using nitrogen gas afterwards. Next, slides were plasma treated for 1 minute. Imaging spacer stickers were added to the cleaned glass slides. Particles were diluted to OD = 0.1 (at 650 nm, 1 cm pathlength) in PBS, and for each sample 50 µL was pipetted into the imaging spacer. Samples were incubated for at least 5 minutes, after which the majority of particles were firmly immobilized. Depending on the measurement, samples were used as described, or briefly washed with PBS (unfolding/refolding measurements).

Fluorescence microscopy measurements

Samples were measured through fluorescence microscopy. The utilized setup's main components consisted of a Coherent OBIS 637 nm laser, Nikon eclipse Ti2 microscope body, Apo TIRF 60x 1.49 NA DIC N2 Oil objective, and Prime BSI Express CMOS camera. All measurements were performed using TIRF illumination. Sample and imaging conditions per experiment are listed in the table below.

Table S.2. Sample and imaging conditions per experiment.

Experiment/associated Figure(s):	Laser power (mW):	Exposure (ms):	Labeled peptide concentration (nM):
Kinetic analysis – dissociation kinetics. Figure 2.C.	50	20	1
Kinetic analysis: association kinetics:. Figure 2D.	50	60	1
PDZ unfolding/refolding experiments. Figure 3.	90	40	2

Peptide and urea solutions were all prepared in PBS. For the PDZ unfolding study at different urea concentrations, a new sample was used for each urea concentration, and measurements were performed with urea present in solution. For the refolding measurement, a sample was pre-incubated with 6.4 M urea for 20 minutes, after which the urea solution was taken off, the sample washed, the peptide solution added, and the sample imaged. All samples were pre-illuminated for several minutes to bleach signals originating from initial a-specific peptide binding events. Durations of pre-illumination steps were kept consistent within the samples belonging to the same experiment.

Supplementary figures

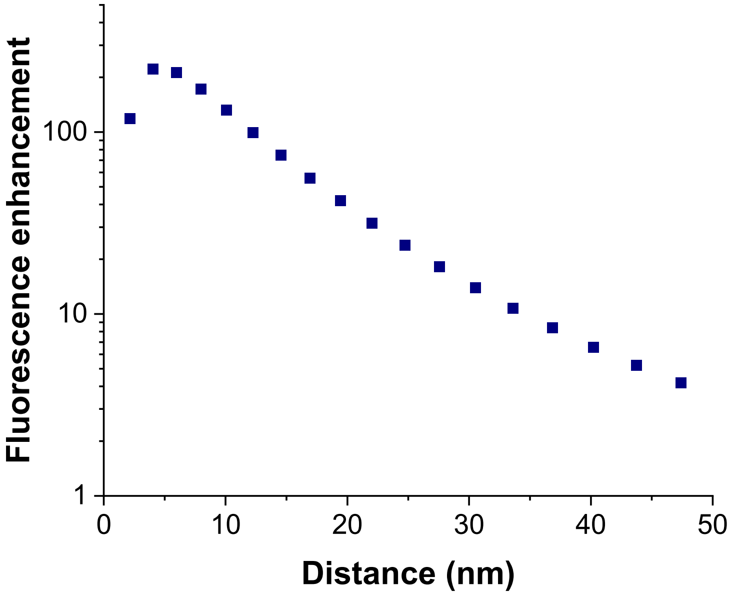

Supplementary Figure 1. Simulated fluorescence enhancement (fold change) of a freely rotating ATTO 655 dye when at a certain distance of (the tip of) a 40x80 nm^2 gold nanorod. The boundary element method was performed using the MNPBEM toolbox, and the simulation combines both the effect of the distant dependent change of the dye's quantum yield, as well as the distance dependent near-field enhancement.

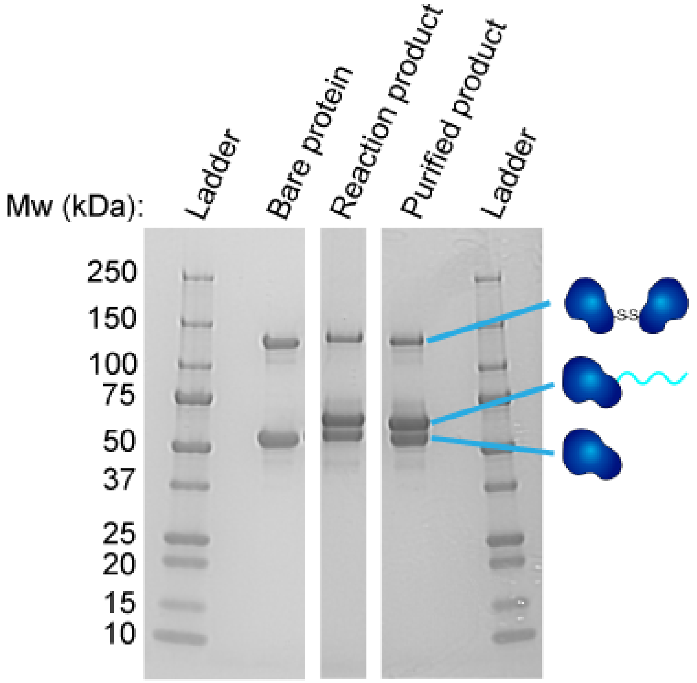

Supplementary Figure 2. SDS-PAGE analysis of the protein-DNA conjugation (Coomassie protein staining, Biorad). No reducing agent added, samples heated at 90 °C for 10 minutes. Lanes from left to right: MW standard proteins bare (no dye) MBP-PDZ, protein-DNA conjugation reaction mixture (post-incubation), final product obtained after Strep-Tactin affinity chromatography and buffer exchange. The final product contains unreacted protein (both singular and disulfide-mediated dimers), which was not removed as it was not predicted to interfere with the protein-GNP

conjugations.

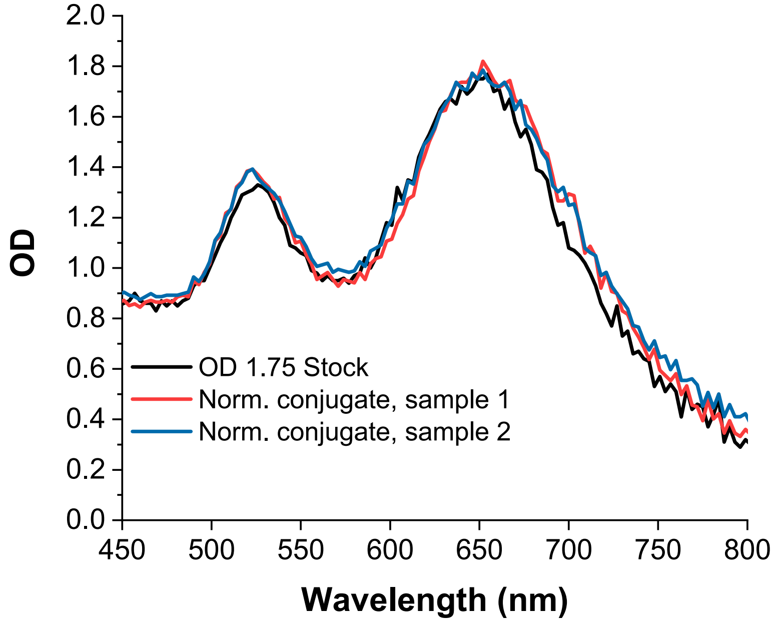

Supplementary Figure 3. Absorption spectra of protein-GNP conjugation products, normalized to a stock solution of bare OD 1.75 particles. The clear spectral overlap showcases the maintained stability of the GNPs. Unnormalized spectra were used to determine the OD values (and thus particle concentrations) of the conjugation products.

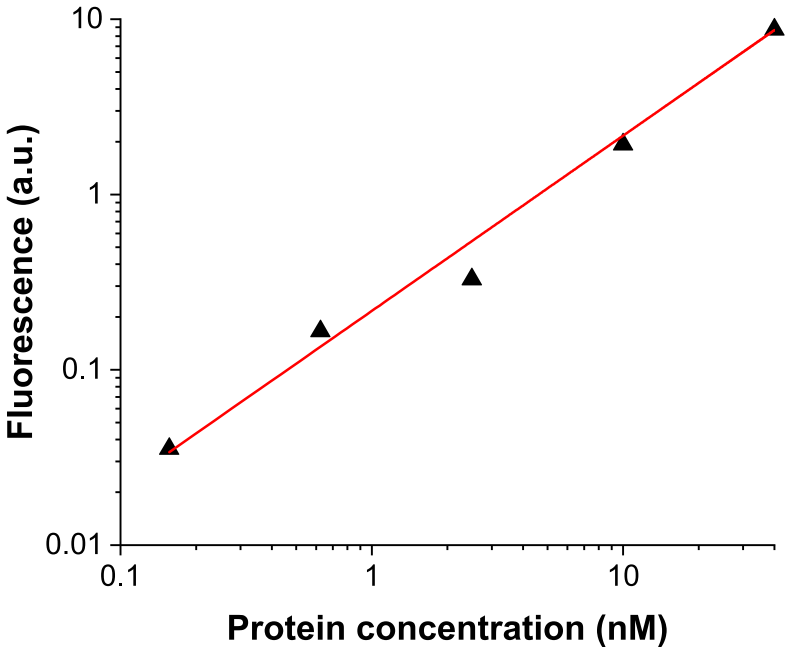

Supplementary Figure 4. Calibration curve used to correlate the measured fluorescence values (at 532 nm) to known concentrations of ATTO 532 labeled MBP-PDZ. Protein concentrations of calibration curve samples determined using a Nanodrop 1000 apparatus, using absorption at 280 nm, corrected for the dyes' absorption at this wavelength. The origin of the linear fit is set to 0.

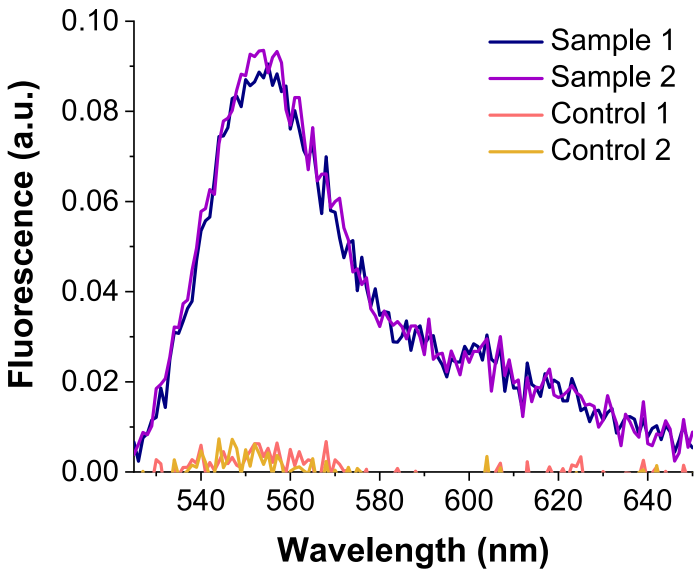

Supplementary Figure 5. Fluorescence spectra of DNA-coated particles incubated with PDZ-DNA (sample 1 & 2), and of DNA-coated particles incubated with bare PDZ (control 1 & 2). Measurements performed on a Varioskan Lux platereader (Thermo Fisher Scientific), in 10 mM HEPES (pH 7.4), 0.0025 w/v% Tween20, 1 mM $MgCl_2$, 0.02 w/v% BSA. The results of a blank measurement (only buffer) were subtracted from all curves.

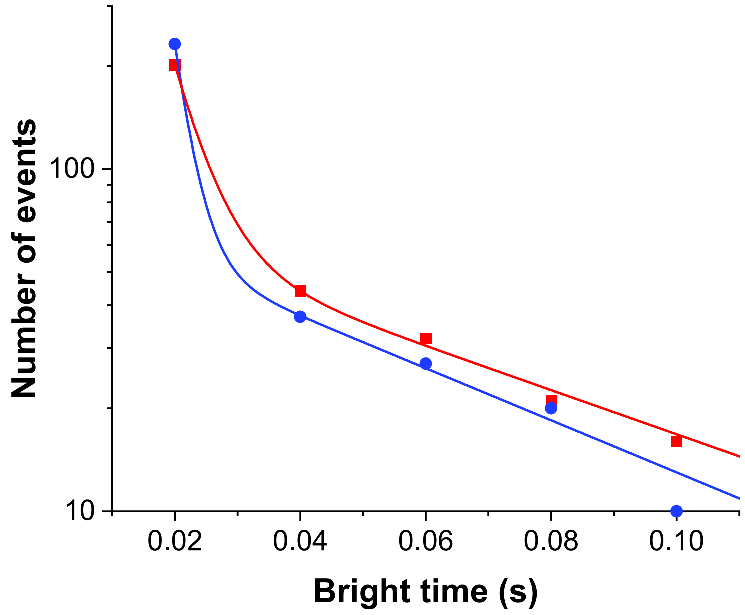

Supplementary Figure 6. Plot depicting the double-exponential distribution of bright times (bound-state lifetimes) of the protein-peptide interactions on two individual PDZ functionalized particles (originating from Figure 2.C's measurement).

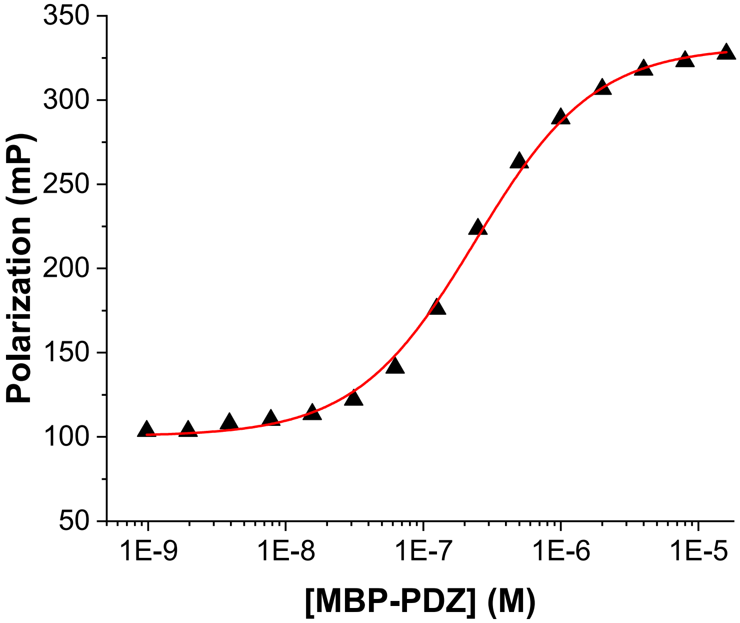

Supplementary Figure 7. Fluorescence polarization curve of the interaction between MBP-PDZ (no DNA attached) and ATTO 655-labeled peptide. The fit $(y = ((A_1 - A_2)/(1 + \left(\frac{[protein]}{K_d}\right)^p) + A_2$, yields a K_d (x_0) of 0.2 µM (p set to 1 to represent 1:1 binding). Measurement performed with 10 nM peptide in 10 mM HEPES pH 7.4, 150 mM NaCl, 0.1 v/v% Tween 20, 0.1 w/v% BSA, 1 mM DTT.

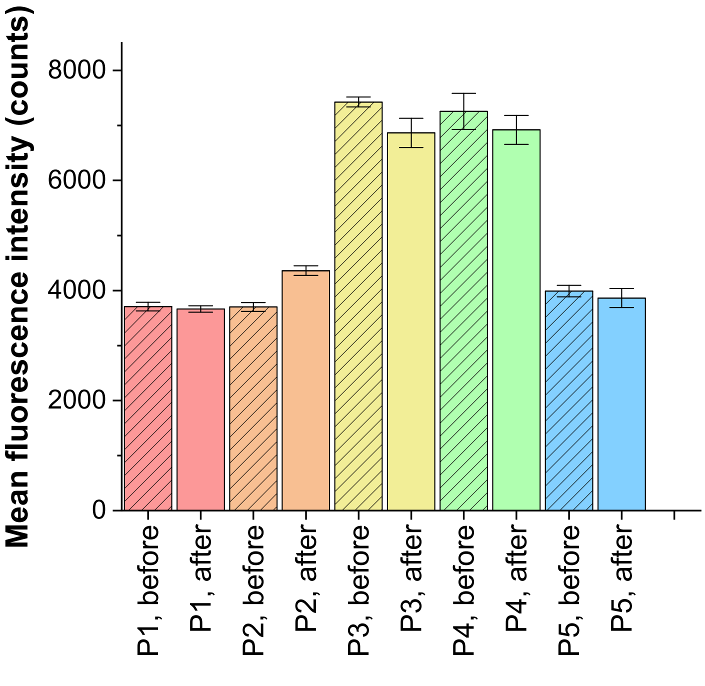

Supplementary Figure 8. Mean fluorescence intensities of five ATTO 532- PDZ decorated gold nanoparticles before and after exposure to 4 M urea. Immobilized particles were briefly imaged with a 532 nm laser before exposure to urea, with bare PBS in the bulk solution. Next, the laser was turned off, urea was added towards 4 M, and the sample was incubated for 15 minutes. Afterwards, the urea solution was exchanged for fresh PBS, the laser turned on, and fluorescence intensities measured again. The particles showed no reduced fluorescence intensities, indicating that the ATTO 532-labeled PDZ proteins remained on the particles.
